# A new setting to improve noninvasive neurally adjusted ventilatory assist by helmet

**DOI:** 10.1186/cc13459

**Published:** 2014-03-17

**Authors:** F Longhini, G Cammarota, C Olivieri, R Perucca, R Vaschetto, D Colombo, A Messina, F Della Corte, P Navalesi

**Affiliations:** 1 Universita del Piemonte Orientale, Novara, Italy

## Introduction

Noninvasive neurally adjusted ventilatory assist by helmet (hNAVA) was shown to improve, compared with pressure support ventilation by helmet (hPSV), patient-ventilator interaction and synchrony in patients with acute respiratory failure without affecting peak electrical activity of the diaphragm (EAdipeak) [1]. Recently, a new helmet is available, which improves pressurization during hPSV. We propose a new setting of hNAVA (hNAVA15) to achieve further improvement. We compare hPSV, hNAVA and hNAVA15, all delivered using the new helmet, with respect to patient's dyspnea, assessed by a visual analogue scale (VASd), arterial blood gases (ABGs), EAdipeak, rate of ventilator pressurization and triggering performance.

## Methods

Fifteen patients underwent three randomized 30-minute trials: hPSV, set with an inspiratory support above positive end- expiratory pressure (PEEP) ≥ 10 cmH_2_O and the fastest rate of pressurization; hNAVA, setting the NAVA level to achieve the same EAdipeak as during hPSV; hNAVA15 setting the NAVA level at 15 cmH_2_O/μV and the maximum inspiratory airway pressure (Paw) at the value corresponding to PEEP + inspiratory support during nPSV. Oxygen inspiratory fraction and PEEP remained unmodified throughout the study period. Paw-time products of the initial 200 ms from the onset of ventilator pressurization (PTP200), of the initial 500 ms from the onset of the EAdi swing indexed to the ideal PTPaw (PTP500i), and of the triggering area (PTPt) were computed. ABGs and VASd were assessed at the end of each trial.

## Results

hNAVA15 reduced the EAdipeak (10.2 (7.1; 16.2) μV) with respect to both hPSV (16.9 (12.7; 19.8) μV *P *< 0.001) and hNAVA (15.3 (10.7; 18.8) μV *P *< 0.001), while decreasing VASd (3.0 (3.0; 4.0) in hPSV, 3. (2.0; 4.0) in hNAVA and 1.0 (1.0; 2.0) in hNAVA15; *P *< 0.01). PTP200 and PTP500i were improved by hNAVA15 (36 (28; 57) cmH_2_O*second and 40 (30; 47)%, respectively) compared with hPSV (31 (24; 45) cmH_2_O*second and 17 (9; 26)%, respectively) and hNAVA (23 (16; 30) cmH_2_O*second and 23 (17; 37)%, respectively) (*P *< 0.01). PTPt was lower in hNAVA15 (2.9 (1.6; 4.4) cmH_2_O*second, *P *< 0.01) compared with both hPSV and hNAVA, and lower in hNAVA (6.0 (2.7; 11.6) cmH_2_O*second), compared with hPSV (18.5 (11.2; 22.5) cmH_2_O*second, *P *< 0.01). ABGs were no different between trials.

## Conclusion

In comparison with hPSV and hNAVA, hNAVA15 significantly reduced EAdipeak and VASd, improving the pressurization and triggering performance, without affecting ABGs.
